# Impact of Music as Environmental Enrichment on Chronic Stress and Anxiety‐Related Responses in Broiler Chickens

**DOI:** 10.1111/asj.70254

**Published:** 2026-10-15

**Authors:** Ana Carla Moreira Cardoso, Jaqueline Murbach Braz, Agnes Markiy Odakura, Caio Cesar dos Ouros, Rodrigo Garófallo Garcia, Maria Fernanda de Castro Burbarelli, Marconi Italo Lourenço‐Silva, Ibiara Correia de Lima Almeida‐Paz, Cláudia Marie Komiyama, Fabiana Ribeiro Caldara

**Affiliations:** ^1^ School of Agrarian Sciences Federal University of Grande Dourados Dourados Mato Grosso do Sul Brazil; ^2^ Department of Animal Production and Preventive Veterinary Medicine, School of Veterinary Medicine and Animal Sciences (FMVZ) São Paulo State University (UNESP) Botucatu São Paulo Brazil

**Keywords:** attention bias, auditory enrichment, corticosterone, poultry production, welfare

## Abstract

This study evaluated the effects of classical music as auditory environmental enrichment on chronic stress and anxiety‐related responses in broiler chickens. Two hundred Cobb 500 chicks were assigned to a control group (no enrichment) or an enriched group exposed to classical music for 6 h daily from 1 to 40 days of age. At 40 days, affective states were assessed using the attention bias test (ABT), while chronic stress was evaluated through feather corticosterone (CORT) concentrations. Music‐exposed birds had significantly lower feather CORT concentrations than controls (*p* < 0.0001), indicating reduced chronic stress. No significant treatment effects were detected for ABT behavioral measures. However, music‐exposed birds showed a numerically longer latency to take the first step (*p* = 0.055), although this difference did not reach statistical significance. Overall, these findings demonstrate that classical music enrichment can reduce physiological indicators of chronic stress in broilers, while evidence for effects on anxiety‐related behavior remains inconclusive. The results suggest that auditory enrichment may help reduce chronic stress in broilers under the conditions evaluated and highlight the need for further replicated studies to clarify its effects on affective states and behavioral responses.

## 1 Introduction

The welfare of broiler chickens has become an increasing concern in industrial poultry production, particularly due to the challenges imposed by intensive rearing systems, which may negatively affect both their physical health and emotional state (Riber et al. 2018; Anderson et al. 2021). Traditionally, animal welfare strategies have prioritized the reduction of negative stimuli, such as stress and anxiety; however, there is a growing recognition of the need to also promote positive experiences throughout the animals' lives, thereby fostering positive affective states. Affective state can be defined as the animals' enduring emotional experience, which shapes their perception of and response to the environment. It results from the balance between positive and negative emotions accumulated over time (Barrett et al. 2007; Kremer et al. 2020) and is influenced by both genetic factors and management conditions (Kremer et al. 2020).

Environmental enrichment has been widely studied as a tool to improve animal welfare, as it provides stimuli that promote the expression of natural behaviors and foster positive emotional states (Jacobs et al. 2023). Among the different forms of enrichment, music has emerged as a practical, low‐cost, and easily applicable strategy, with the potential to modulate stress responses and influence the animals' emotional perception (Papadakakis et al. 2019; Fu et al. 2023).

To assess the impact of auditory enrichment on broiler welfare, it is essential to adopt methodologies that quantify both chronic stress and affective states. One of the most commonly applied physiological approaches in this context is the quantification of corticosterone (CORT) in feathers, a biomarker that reflects prolonged stress exposure throughout the growth period. However, glucocorticoids primarily indicate arousal or the intensity of a stimulus, rather than its valence (Boissy et al. 2007; Staley et al. 2018). Indeed, positive and negative stimuli of equal intensity may elicit indistinguishable glucocorticoid responses (Otten et al. 2002). Therefore, while feather CORT provides valuable information about the cumulative activation of the hypothalamic–pituitary–adrenal (HPA) axis and chronic stress burden, it cannot by itself confirm improvements in affective state and must be interpreted in conjunction with additional physiological, behavioral or cognitive measures.

The assessment of affective state may be achieved using validated cognitive approaches, among which the attention bias test (ABT) has been widely applied. This test is based on the premise that animals experiencing negative emotional states, such as fear or anxiety, are more predisposed to detect and respond to potentially threatening stimuli in their environment. Accordingly, the ABT assesses attentional focus by quantifying vigilance behaviors (e.g., alertness, freezing) that animals allocate toward a specific stimulus (threatening or negative) in comparison to another (non‐threatening or positive) (Crump et al. 2018). Attention bias is closely associated with anxiety, and research indicates that anxious individuals generally devote more attention to negative stimuli than calmer individuals (Eysenck et al. 1991; Mogg and Bradley 1998). Thus, the test provides an indirect measure of how the rearing environment influences the animals' emotional perception.

Based on these premises, the present study aimed to evaluate the effects of continuous exposure to music on the welfare of broiler chickens, using feather CORT quantification and the ABT as tools to measure chronic stress and affective states. Our hypothesis is that music, as a form of environmental enrichment, can modulate the birds' emotional state, reducing stress and promoting more positive emotions throughout the production cycle.

## 2 Materials and Methods

The experiment was carried out at the Federal University of Grande Dourados (UFGD), MS, Brazil. The experimental protocol was approved by the Animal USE Ethics Committee of UFGD (number 01/2022 CEUA) and all procedures were performed in accordance with relevant guidelines and regulations.

### 2.1 Chickens, Facilities, and Management

Two hundred one‐day‐old male Cobb 500 chicks from a commercial hatchery were used. The trial was carried out in two small and identical climate‐controlled poultry barns (6 × 1.50 m) with negative pressure ventilation. Both barns were provided with 10 cm of new wood shavings as bedding, three hanging galvanized tube feeders, and three semi‐automatic bell drinkers. Each barn contained 100 chickens and a targeted maximum stocking density of 37 kg/m^2^. The litter was turned out weekly and new wood shavings were added as needed. Both barns' temperatures were gradually decreased from 32°C on Day 1 to 21°C on Day 36 and remained at 21°C until Day 42. These parameters were monitored daily using a data logger (Elitech RC‐51 USB, Brazil) positioned at the center of each barn, at a height of 30 cm above the litter. The chickens were maintained on an artificial lighting program of 24L:0D during the first 7 days due to the heat lamps. From Day 8 onwards, the lighting was adjusted to 18L:6D until Day 14, and then to 16L:8D until the end of the trial, following the recommendations of the Cobb management guide.

The corn–soy diets were prepared according to the nutritional specifications for conventional broiler chickens (Rostagno 2017) and were separated into five rearing phases: pre‐starter (1–7 days, 25.3% CP and 3000 kcal ME/kg), starter (8–21 days, 24.3% CP and 3100 kcal ME/kg), grower I (22–33 days, 22.6% CP and 3200 kcal ME/kg), and grower II (34–42 days, 19.5% CP and 3250 kcal ME/kg). Both feed and water were provided ad libitum.

### 2.2 Experimental Design

Chickens were randomly allocated to either music enrichment or control treatment (*n* = 100 chickens/treatment). Each treatment was housed in a single, separate barn (one barn per treatment), so that treatment and barn were confounded and the barn served as the experimental unit for treatment comparison, a design aspect that is further considered in the Discussion.

The barn for the control treatment provided an environment similar to that used in commercial broiler husbandry. The barn for the music enrichment treatment had the same setup as the control barn, with the addition of a portable Bluetooth speaker (Frahm—model Lumi Tower 900, Brazil) placed at 1.5 m height in the center of the barn to play the music enrichment protocol at approximately 75 dB. Sound pressure was monitored throughout the entire facility using a digital sound level meter (AKSO—model KR813, Brazil), ensuring a homogeneous distribution of sound and guaranteeing that the levels did not exceed the recommended limit. In both barns, background sound pressure levels in the absence of music typically ranged between 30 and 65 dB, mainly due to ventilation and other equipment and occasional external noises; thus, the primary acoustic difference between treatments was the presence of the classical music playback in the enrichment barn.

The music enrichment protocol consisted of daily exposure to 6 h of music divided into three periods of 2 h each, interspersed with 2‐h silent intervals, starting at 07:00 h, beginning on the day of placement when chicks were 1 day old and continuing until the end of the experimental period at 40 days of age (i.e., 39 days of treatment). A playlist of music considered to have a moderate tempo, based on suites, symphonies, and concertos with Andante (75–107 BPM) and Andante Moderato (90–100 BPM) tempos, composed for cello, violin, and string instruments (including Bach—Suite No. 1 for Cello in G Major, Bach—Violin Concerto in A Minor, Mendelssohn—String Symphony No. 4, Bach—Brandenburg Concerto No. 4 in G, Bach—Brandenburg Concerto No. 1 in F, Bach—English Suites, and Mozart—Symphony No. 33 in B‐flat Major) was used. The same playlist was played every day throughout the experimental period, and tracks were always presented in the same fixed order within each 2‐h session.

The acoustic characteristics of the playlist, including its frequency composition and sound intensity, are illustrated in Figure 1. The horizontal axis represents sound frequency (Hz), while the vertical axis represents sound intensity (dB), with values closer to 0 dB indicating greater sound energy. Each bar represents the amount of acoustic energy present at a given frequency. Higher peaks therefore indicate frequencies that were more strongly represented in the playlist. Most of the acoustic energy was concentrated between approximately 63 and 130 Hz, corresponding to the fundamental frequencies of the cello and violin, whereas higher frequencies mainly represent harmonic components of these instruments.

**FIGURE 1:**
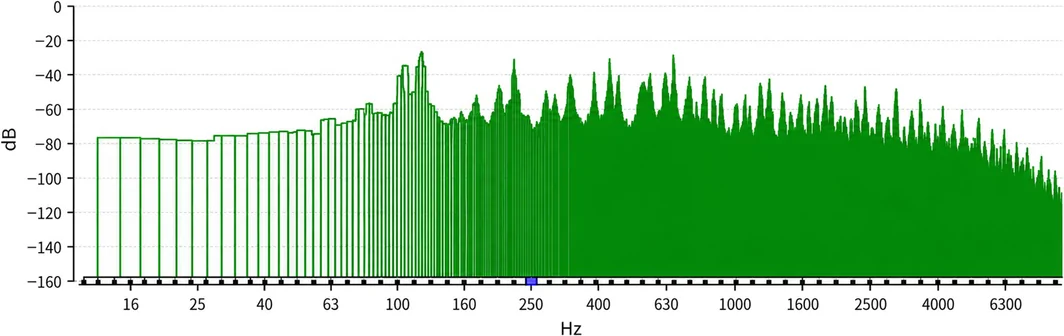
Representative power spectrum of the music enrichment playlist. The x‐axis shows sound frequency (Hz), and the y‐axis shows sound intensity (dB). Higher peaks indicate frequencies with greater acoustic energy. Most of the energy is concentrated between approximately 63 and 130 Hz, corresponding to the fundamental frequencies of the string instruments used in the selected repertoire.

### 2.3 Measurements

#### 2.3.1 ABT

At 40 days of age, the ABT was conducted at the group level by a single trained observer, following a modified method as described in Anderson et al. (2021) and pharmacologically validated in Lourenço‐Silva et al. (2024). Testing was conducted in a square wooden arena (1.40 m × 1.40 m × 0.90 m) containing pine shavings as bedding and a trough feeder with familiar feed and mealworms as a positive stimulus. Two cameras were positioned overhead and lateral to record live and later behavioral responses, respectively (Figure 2). A portable Bluetooth speaker was placed near the arena to play a conspecific ground predator alarm call for 8 s, as a negative stimulus, at 72 dB. To prevent the conspecific alarm call to be heard in the experimental barns, the arena was placed in an enclosed separate room 100 m away.

**FIGURE 2:**
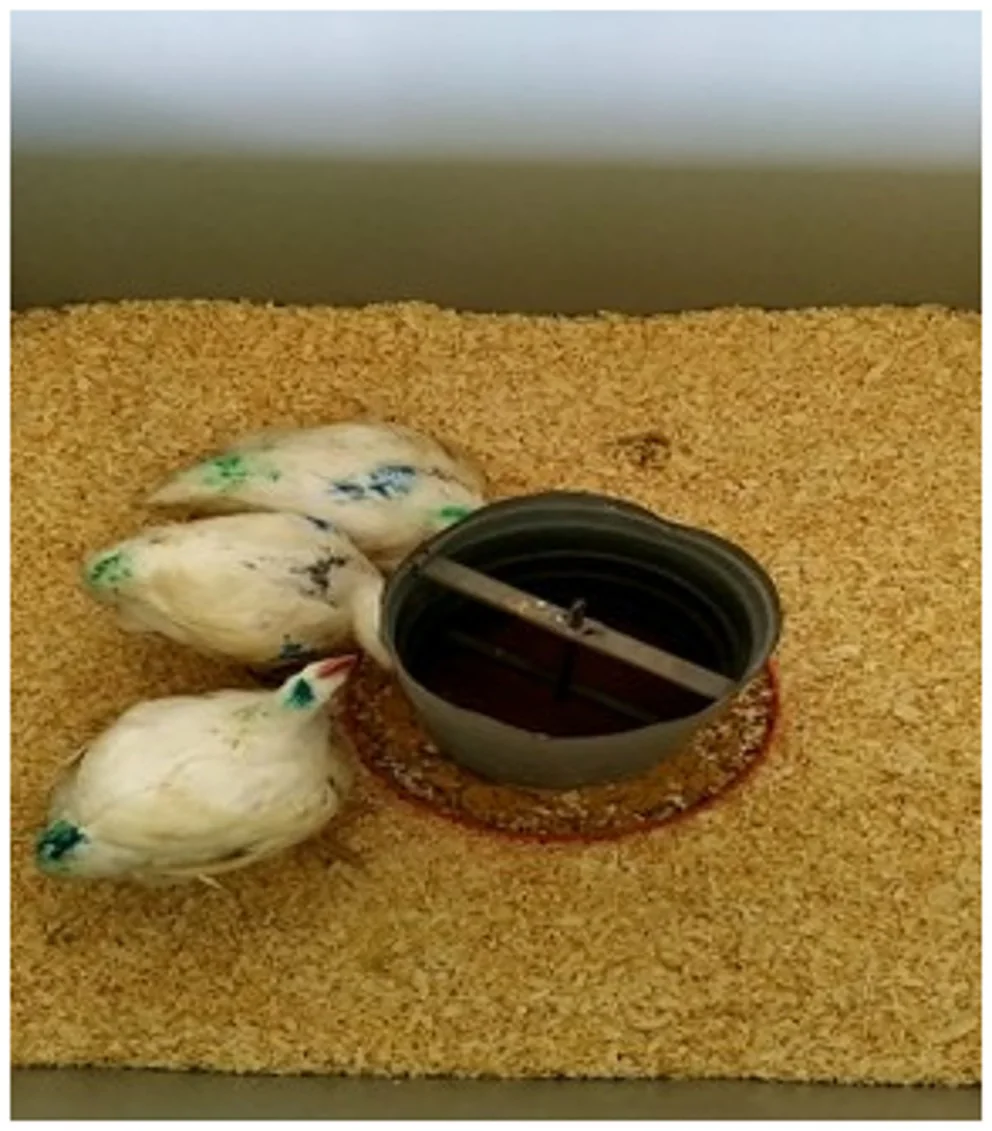
Experimental arena for the attention bias test. Representative video frame showing the experimental arena containing wood shavings as bedding and a trough feeder containing familiar feed and mealworms. The test was recorded using two cameras positioned overhead and laterally.

#### 2.3.2 Attention Bias Procedures

Testing was performed using groups of three birds (*n* = 7 groups of three birds/treatment), with each group tested only once. Because birds tested simultaneously could potentially influence one another's behavioral responses during the test, each group of three birds was defined as a testing round. Testing round was subsequently included as a random effect in the statistical models to account for the potential lack of independence among birds evaluated simultaneously within the same test group. Although birds were tested in groups of three, ABT behavioral variables were recorded at the bird level (*n* = 21 birds/treatment). Behavioral variables were analyzed at the bird level, except that latency to first vocalization included only birds that vocalized during the observation period. To avoid any influence of repeated handling or prior testing on ABT responses, the birds selected for the ABT and feather sampling were not used in any other tests. The sample size for the ABT was determined a priori using a power analysis, which indicated that seven groups of three birds per treatment were sufficient to detect meaningful treatment differences while maintaining birds naïve to other experimental procedures.

To ensure that walking ability did not confound behavioral responses, gait scoring was performed 1 day before testing, at 39 days of age, as described in Dawkins et al. (2004), and only birds with a gait score of 0 were included in the test. At this time, birds were individually identified using non‐toxic colored marker sticks applied to different body regions (head, wings, and back), allowing reliable identification of each bird during the subsequent behavioral tests.

On the testing day, previously selected chickens (*n* = 21 chickens per treatment) were transported to two holding pens (3.14 m^2^ each) in an adjacent room to allow acclimation and reduce transport stress. The holding pens replicated home‐pen conditions, containing familiar conspecifics, pine shavings, feed and water. Groups of three chickens were then randomly assembled using body markings for identification.

During testing, the observer remained in the testing room but outside the chickens' visual field and was blinded to the treatments. Additionally, video recordings were coded to ensure blinding during subsequent behavioral analyses. Live behavioral response was recorded via a monitor connected to the overhead camera and included latency to begin feeding (s). Late behavioral responses were analyzed from video recordings and included latency to first vocalization (s), latency to first step (s), and the occurrence (yes/no) of vigilance behaviors (freezing, stretching the neck, looking around, or adopting an erect posture) within the first 30 s following the alarm call (Anderson et al. 2021). For latency to first vocalization, only birds that vocalized during the observation period contributed to the analysis, as a latency value could not be determined for birds that did not vocalize.

#### 2.3.3 Chronic Stress (Feather CORT)

After ABT (Day 40), three wing feathers were cut from all tested chickens to determine feather CORT concentrations (*n* = 21 birds/treatment). Feathers were collected by cutting the calamus as close to the skin as possible, avoiding any contact or damage to the skin. After collection, feathers were placed in sterile plastic bags and stored at −80°C until analysis.

Feather CORT concentrations were determined following the methanol‐based extraction procedure described by Bortolotti et al. (2008), adapted for broilers, using a fixed feather mass and extraction volume for all samples. Briefly, feathers from each bird were minced into pieces smaller than 5 mm and thoroughly homogenized. A constant mass of 10 mg of feather material was weighed for each sample and placed into 20 mL scintillation vials containing 1 mL of HPLC‐grade methanol. Vials were placed in a sonicating water bath for 30 min at room temperature and then incubated overnight in a shaking water bath at 56°C to facilitate steroid extraction.

After incubation, samples were filtered to remove feather material, and the filtrate was transferred to clean vials. Methanol was then allowed to evaporate completely under a fume hood. The dried extract was reconstituted in 1 mL of ELISA buffer supplied with the commercial kit. Reconstituted CORT concentrations (ng/mL) were quantified in duplicate using a commercial ELISA kit (Abcam, Cambridge, MA, USA) according to the manufacturer's protocol. The resulting CORT concentrations were normalized to the mass of feather material extracted and expressed as pg/mg of feather. The assay characteristics (specificity, parallelism, spike‐and‐recovery, and sensitivity) have been validated in these earlier studies (Carbajal et al. 2014; Häffelin et al. 2020), and therefore a full validation was not repeated here. Intra‐assay coefficients of variation for our samples were 3.4%, indicating acceptable analytical precision. All samples fell within the assay's reliable detection window (20%–80% B/Bo).

### 2.4 Statistical Analysis

Data were analyzed in SAS Studio 3.8 (SAS Institute Inc., Cary, NC, USA). Homogeneity of variances was assessed using Levene's test, and data residuals' normality was verified by the Shapiro–Wilk test. Bird was considered the observational unit for all response variables. For latency to first vocalization, the analysis included only birds that vocalized during the observation period, as described above. Normally distributed dependent variables included ABT Latency to first vocalization (s), latency to first step (s), and feather CORT concentrations (pg/mg). Dependent variables without normally distributed residuals included ABT Latency to begin feeding (s) and birds (%) displaying vigilance behaviors. Generalized linear mixed models (GLIMMIX) were applied for latency to begin feeding data using lognormal distribution and for latency to first vocalization and latency to first step data using Gaussian distribution, followed by *F*‐tests with treatment as a fixed effect and testing round as a random effect. The inclusion of testing round as a random effect accounted for the potential correlation among birds exposed to the same testing environment and alarm‐call stimulus during a single ABT session. Denominator degrees of freedom were determined according to the approximation implemented in PROC GLIMMIX and may therefore differ among response variables depending on the fitted model and variance–covariance structure. The data are presented as LS means ± SEM. For the variable analyzed on the log scale (latency to begin feeding), LS means were back‐transformed to the original scale (s) for presentation.

Vigilance behaviors (freezing, neck stretching, erect posture, and looking around) were coded as binary variables (0 = absence, 1 = presence within 30 s after the alarm call) and assessed by GLIMMIX using binary distribution, followed by *F*‐tests with treatment as a fixed effect and testing round as a random effect. The data are presented as descriptive percentages obtained using the FREQ procedure. Feather CORT concentrations data were subjected to ANOVA using the MIXED procedure, followed by *F*‐test, considering a significance of *p* ≤ 0.05.

## 3 Results

### 3.1 ABT

Latency to take the first step was longer for chickens in the music enrichment treatment (15.4 ± 2.3 s) than for birds in the control treatment (9.8 ± 2.2 s; F_1,24_ = 4.08; Figure 3), although this difference did not reach conventional statistical significance (*p* = 0.055).

**FIGURE 3:**
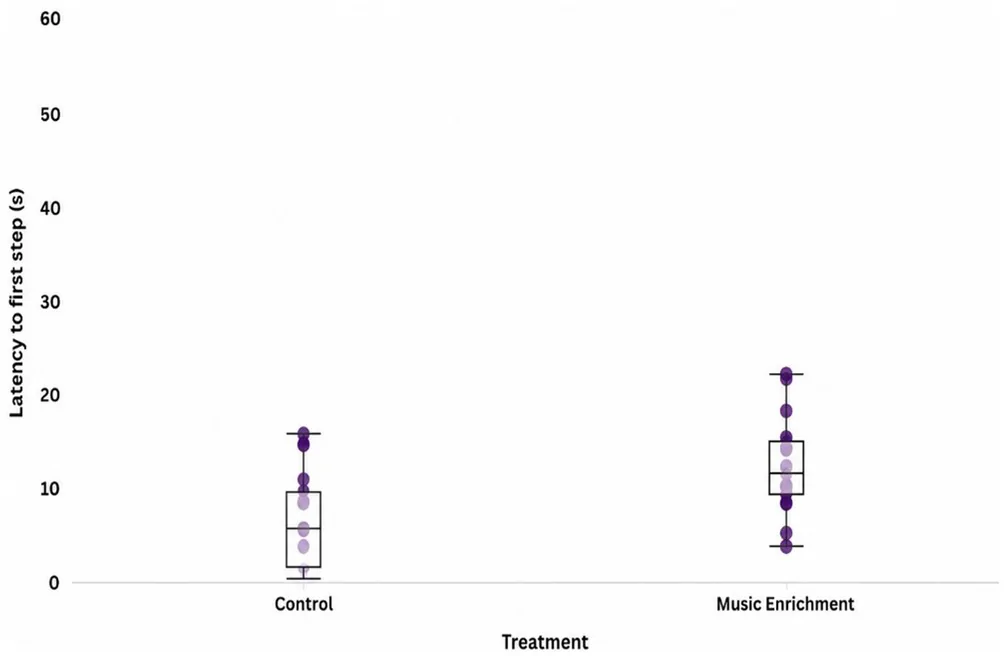
Boxplot for least square mean estimates (mean ± SEM) of latency to first step for broiler chickens from control and enriched treatments. The treatment effect was not statistically significant (*p* = 0.055). SEM = standard error of the mean.

There was no statistically significant effect of music on latency to begin feeding in the ABT (F_1,39_ = 3.22; *p* = 0.080), although birds in the music enrichment treatment showed a shorter latency to begin feeding (14.9 ± 2.5 s) than those in the control treatment (21.0 ± 3.5 s; Figure 4).

**FIGURE 4:**
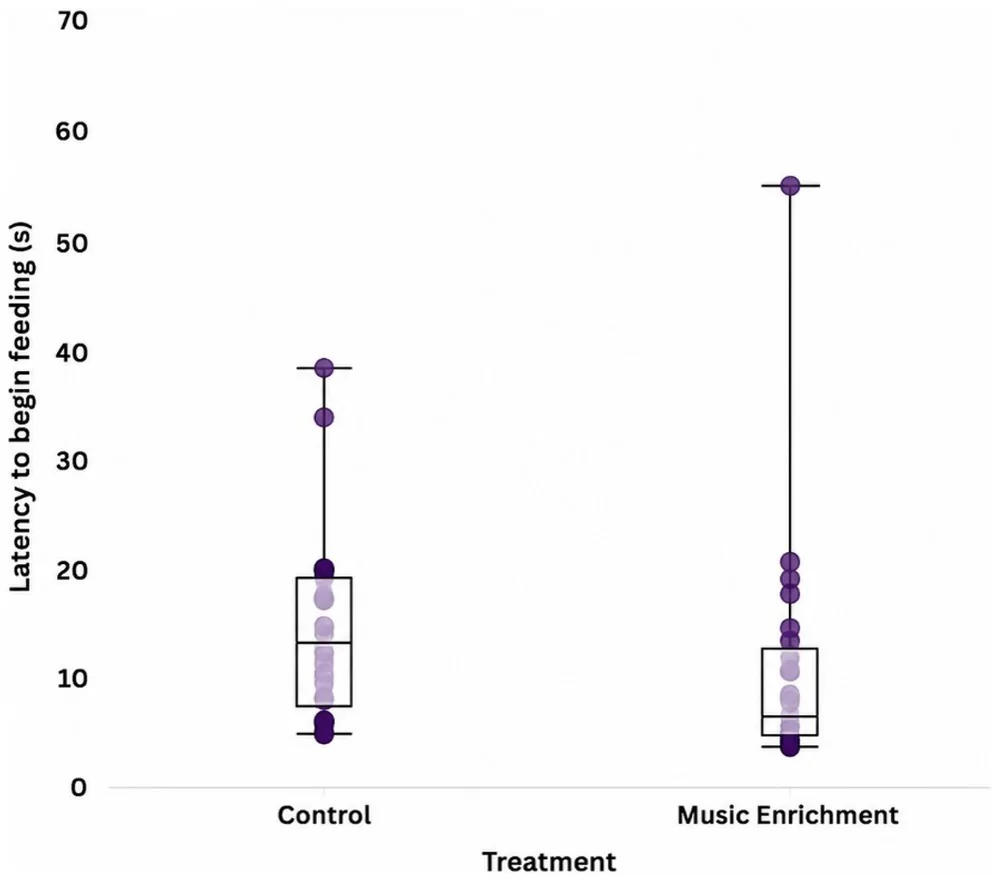
Boxplot for least square mean estimates (mean ± SEM) of latency to begin feeding for broiler chickens from control and music enrichment treatments. The treatment effect was not statistically significant (*p* = 0.080). SEM = standard error of the mean.

The latency to first vocalization did not differ between treatments (F_1,14_ = 2.57; *p* = 0.169). Similarly, the occurrence of vigilance behaviors did not differ between treatments (*p* ≥ 0.717; Table 1).

**TABLE 1:** Least square mean for latency to first vocalization (s) and proportion (%) of total chickens performing vigilance behaviors from either control or music enrichment treatments at 40 days of age.

Behavior	Treatment		Test statistic and *p*
	Control	Enriched	
Latency to first vocalization (s)	7.5	14.4	F_1,14_ = 2.57; *p* = 0.169
Neck stretching (% of birds)	64.0	66.7	F_1,39_ = 0.03; *p* = 0.860
Erect posture (% of birds)	24.0	29.2	F_1,39_ = 0.11; *p* = 0.737
Looking around (% of birds)	81.5	85.2	F_1,44_ = 0.13; *p* = 0.717
Freezing (% of birds)	92.0	100	F_1,47_ = 0.01; *p* = 0.979

### 3.2 Chronic Stress (Feather CORT)

Feather CORT concentrations by feather weight were higher in chickens from the control treatment (36.9 ± 0.6 pg/mg) than those from the enriched treatment (26.7 ± 0.9 pg/mg; F_1,41_ = 101.0; *p* < 0.001, Figure 5).

**FIGURE 5:**
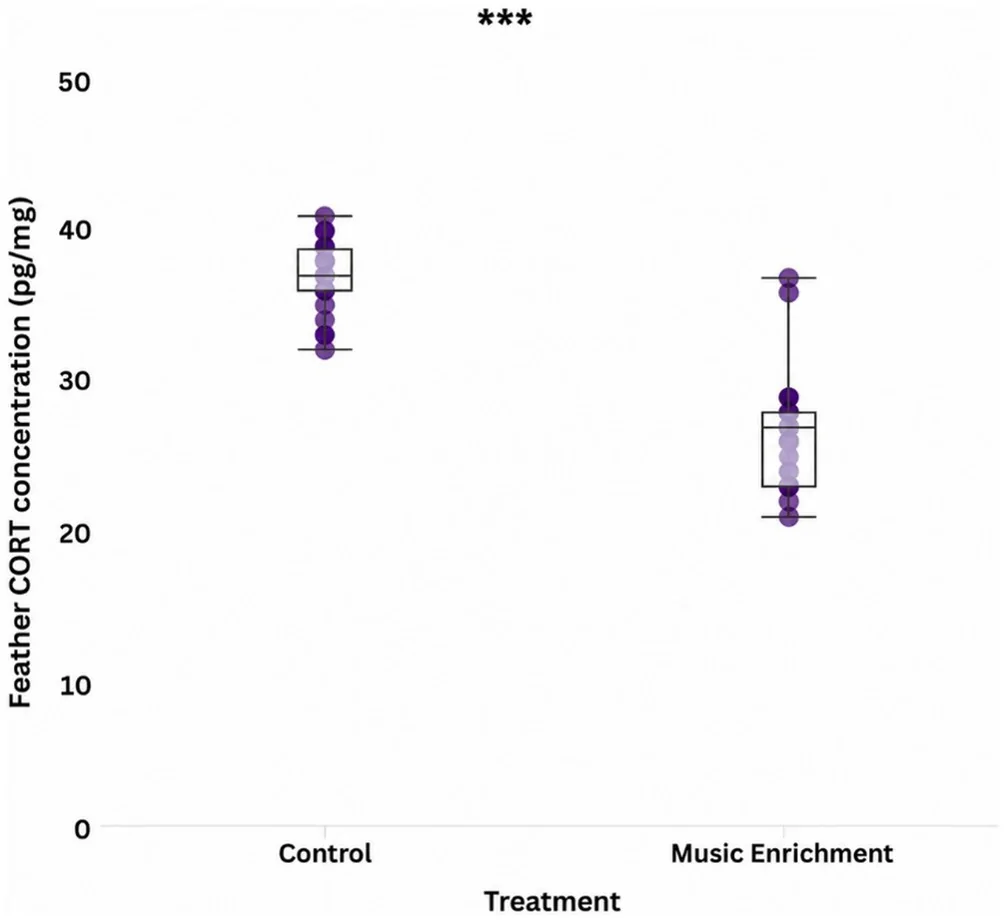
Least square mean estimates (± SEM) of the total feather corticosterone (CORT) concentrations for broiler chickens from control and enriched treatments. *** indicates a difference at *p* ≤ 0.001.

## 4 Discussion

In the present study, broilers exposed to music daily showed lower CORT concentrations in feathers at 40 days, consistent with a lower chronic stress burden throughout the production cycle. In contrast, no statistically significant differences between treatments were detected for any of the behavioral measures assessed in the ABT. After the alarm signal, music‐exposed broilers exhibited a longer latency to take the first step than controls (*p* = 0.055), but this numerical difference did not reach conventional statistical significance and should therefore be interpreted as statistically inconclusive. A longer latency to first step may reflect an initial orienting or freezing response, delayed movement, or altered arousal; thus it does not, by itself, provide clear evidence of a positive welfare effect.

Pharmacological validation studies provide a useful context for interpreting this behavioral pattern. In the ABT for fast‐growing broilers, anxiogenic treatment increased the latency to begin feeding and reduced the latency to take the first step relative to controls, demonstrating that delayed feeding is a reliable indicator of heightened anxiety, whereas earlier stepping reflects rapid arousal or escape‐oriented behavior rather than calm vigilance (Lourenço‐Silva et al. 2024). One possible interpretation is that this combination reflects a two‐stage response, with an initial orienting or freezing pause at threat onset, followed by relatively rapid disengagement from the potential threat and reallocation of attention toward the appetitive cue. However, the specific meaning of the longer stepping latency remains uncertain, and alternative explanations (e.g., altered arousal or decision‐making) cannot be ruled out.

In the present study, music‐exposed broilers also showed numerically shorter feeding latencies while displaying a slightly longer latency to take the first step after the alarm call. Because neither behavioral difference reached conventional statistical significance, this pattern should not be interpreted as evidence of reduced anxiety or improved welfare. The observed reduction in feather CORT is consistent with a lower chronic stress burden, whereas the behavioral findings remain statistically inconclusive.

Glucocorticoids such as cortisol and CORT are central mediators of the stress response via the HPA axis (Lightman et al. 2020). While acute elevations of these hormones are adaptive and facilitate coping with challenges, chronic activation of the HPA axis is associated with persistently elevated levels, impaired immunity, reduced growth and reproduction, and the development of negative affective states such as anxiety or anhedonia. Feather CORT is widely recognized as a reliable biomarker of chronic stress in poultry (Romero and Fairhurst 2016; Lim et al. 2025). The lower concentrations observed in the present study therefore suggest that music exposure was associated with a reduced chronic stress burden in broilers, which is consistent with previous findings showing decreased CORT or ACTH under auditory enrichment (Gao et al. 2023; Zhao et al. 2023).

The underlying mechanisms may involve central effects of music on the HPA axis. Musical stimulation has been shown to influence hypothalamic activity, modulate CRH release, and reduce pituitary ACTH secretion, ultimately lowering adrenal CORT output (Menon and Levitin 2005). Moreover, music promotes the release of neurotransmitters linked to well‐being, such as dopamine and serotonin, while reducing sympathetic arousal (Panksepp 1986; Thoma et al. 2013; dos Santos Lemes Lechuga et al. 2023; Oliveira et al. 2024). These neuroendocrine effects provide a plausible explanation for the reduced CORT concentrations detected in music‐exposed birds.

Interestingly, these results also parallel findings in humans. Langer, Jentsch, and Wolf (Langer et al. 2022) demonstrated that elevated cortisol can facilitate the cognitive regulation of high‐intensity negative emotions, particularly through distraction. Although poultry do not deliberately employ cognitive strategies such as reappraisal or distraction, both widely recognized in humans (Webb et al. 2012), the observed effects of music enrichment may be functionally analogous. Although such mechanisms could theoretically influence attentional processing and affective state, the present ABT results did not demonstrate statistically significant behavioral differences between treatments. Therefore, the current findings do not allow conclusions regarding whether music altered attentional allocation or promoted a more positive affective state.

Beyond neuroendocrine mechanisms, music enrichment may influence stress physiology by modifying the acoustic environment. Continuous classical music may have masked sudden or unpredictable noises from the surroundings, ventilation, and feeding equipment, reducing the salience of aversive acoustic events and associated stress (Coleman et al. 2017; Clark and Dunn 2022). Thus, the reduction in feather CORT may reflect both music‐induced emotional modulation and exposure to a more predictable, less noisy soundscape.

Finally, the broader literature supports the role of enrichment in improving cognition and emotion in animals. Studies have shown that enriched environments reduce fear and anxiety (Tahamtani et al. 2015; Ulans et al. 2024), enhance adaptation to challenges (Campbell 2024), and even promote neuroplasticity in the hippocampus (Patzke et al. 2009; Dávila et al. 2011; Lippi et al. 2022). In poultry, auditory enrichment from early stages reduces fear and promotes feeding under stress (Dávila et al. 2011; Korsos et al. 2019), while classical and modern relaxing music has been linked to reduced aggression, lower glucocorticoid levels, and improved productive performance (Gvaryahu et al. 1989; Gao et al. 2023; Ciborowska et al. 2025). Collectively, the findings from these previous studies highlight that music and other forms of enrichment can mitigate stress and may promote positive emotional states and welfare in broiler chickens, even though in the present experiment we did not detect significant effects on behavioral measures of emotional state.

Under the conditions of the present study, daily exposure to classical music as auditory enrichment was associated with a clear reduction in feather CORT concentrations in broiler chickens, consistent with a lower chronic stress burden across the production period.

### 4.1 Limitations of the Study

A key limitation of the present study is the experimental design, as only two barns were used, with one barn assigned to the control treatment and the other to the music enrichment treatment. Consequently, the effect of treatment is confounded with the effect of barn, and we cannot statistically disentangle whether the observed differences, particularly in feather CORT concentrations and attention bias responses, are due solely to the auditory enrichment or partially to unmeasured barn‐specific factors. Although the barns were identical in size, structure, equipment, and environmental management (temperature, ventilation, lighting, litter handling, and husbandry routines), and the same staff cared for both flocks, the lack of true replication at the barn or pen level requires caution when interpreting and generalizing these findings. Future studies should include multiple pens or barns per treatment, ideally with random allocation and rotation of treatments to confirm the robustness and generalizability of the effects of music enrichment on broiler welfare. It is also possible that birds partially habituated to the musical stimulus over time, as the same playlist and fixed track order were used throughout the trial. We did not quantify temporal changes in responsiveness to music, and therefore cannot determine whether its effects on stress physiology were stronger early or late in the production cycle. Future studies could address this by comparing fixed versus varied playlists or by systematically assessing responses at multiple time points.

## Author Contributions

Conceptualization: F.R.C., A.M.O., and J.M.B. Methodology: F.R.C., M.I.L‐S., and A.C.M.C. Formal analysis: M.F.C.B. and M.I.L‐S. Investigation: F.R.C., A.M.O., J.M.B., C.C.O., R.G.G., C.M.K., M.F.C.B., and I.C.L.A‐P. Writing – original draft: F.R.C. and A.C.M.C. Writing – review and editing: M.I.L‐S, J.M.B., C.M.K., C.C.O., and M.F.C.B. Data curation, visualization, and resources: F.R.C. and R.G.G. Funding acquisition: F.R.C., R.G.G., and I.C.L.A‐P. Supervision: F.R.C. and R.G.G. Project administration: F.R.C.

## Funding

Research project financed by the Fundação de Apoio ao Desenvolvimento do Ensino, Ciência e Tecnologia do Estado de Mato Grosso do Sul (FUNDECT), Brazil (Grant No. 71/044.550/2022).

## Conflicts of Interest

The authors declare no conflicts of interest.

## Data Availability

The data that support the findings of this study are available from the corresponding author upon reasonable request.
